# Antimicrobial Properties of α-MSH and Related Synthetic Melanocortins

**DOI:** 10.1100/tsw.2006.227

**Published:** 2006-10-02

**Authors:** A. Catania, G. Colombo, C. Rossi, A. Carlin, A. Sordi, C. Lonati, F. Turcatti, P. Leonardi, P. Grieco, S. Gatti

**Affiliations:** ^1^ Center for Preclinical Investigation, Fondazione IRCCS Ospedale Maggiore Policlinico, Mangiagalli e Regina Elena, Milano 20122, Italy; ^2^ Department of Pharmaceutical Chemistry and Toxicology, Università di Napoli, Napoli 80131, Italy; ^3^ Center for Surgical Research, Fondazione IRCCS Ospedale Maggiore Policlinico, Mangiagalli e Regina Elena, Milano 20122, Italy

**Keywords:** natural antimicrobial peptides, α-melanocyte stimulating hormone, melanocortins, cyclic AMP, Candida albicans, Staphylococcus aureus, neuroimmunomodulation

## Abstract

The natural antimicrobial peptides are ancient host defense effector molecules, present in organisms across the evolutionary spectrum. Several properties of α-melanocyte stimulating hormone (α-MSH) suggested that it could be a natural antimicrobial peptide. α-MSH is a primordial peptide that appeared during the Paleozoic era, long before adaptive immunity developed and, like natural antimicrobial molecules, is produced by barrier epithelia, immunocytes, and within the central nervous system. α-MSH was discovered to have antimicrobial activity against two representative pathogens, *Staphylococcus aureus* and *Candida albicans*. The candidacidal influences of α-MSH appeared to be mediated by increases in cell cyclic adenosine monophosphate (cAMP). The cAMP-inducing capacity of α-MSH likely interferes with the yeast's own regulatory mechanisms of this essential signaling pathway. It is remarkable that this mechanism of action in yeast mimics the influences of α-MSH in mammalian cells in which the peptide binds to G-protein-linked melanocortin receptors, activates adenylyl cyclase, and increases cAMP. When considering that most of the natural antimicrobial peptides enhance the local inflammatory reaction, the anti-inflammatory and antipyretic effects of α-MSH confer unique properties to this molecule relative to other natural antimicrobial molecules. Synthetic derivatives, chemically stable and resistant to enzymatic degradation, could form the basis for novel therapies that combine anti-inflammatory and antimicrobial properties.

## NATURAL ANTIMICROBIAL PEPTIDES

Natural antimicrobial peptides are ancient host defense effector molecules, present in organisms across the evolutionary spectrum[1,2]. The existence of homologues of vertebrate antimicrobial peptides in invertebrates suggests that such agents are ancestral components of the host defense system. Survival of plants and invertebrates, which lack adaptive immunity, illustrates effectiveness of host defense based on such innate mechanisms. These molecules have complex and specific mechanisms of action, within and beyond conformational classes.

Antimicrobial peptides are integral components of the innate defense. They are produced by many tissues and cell types in a variety of animal, plant, and invertebrate species[1,2]. Mucosal secretions, phagocytes, and other components of the innate host defense system initiate the response to microbial penetration before time-consuming adaptive immunity starts. The natural antimicrobial peptides are generally effective against bacteria, fungi, and viruses. Some of these molecules might be used as therapeutic agents to provide microbicidal activity against a broad spectrum of pathogens and/or to enhance effects of the current antimicrobial agents.

Of interest, it appears that mammalian antimicrobial proteins have multiple effects and contribute to both innate and adaptive immunity. For instance, mammalian defensins and cathelicidins also exert several receptor-mediated effects on immune cells[3]. Defensins are chemotactic for T lymphocytes[3]; the human cathelicidin LL-37 is a chemoattractant for T cells, monocytes, and neutrophils through its interaction with the formyl-methionine-leucine-phenylalanine (fMLP) receptor[4]. Further, defensins stimulate mast cell degranulation, leading to increased vascular permeability, neutrophil accumulation, and induction of epithelial synthesis of chemokines. Local inflammatory cascade is amplified by the stimulatory effects of defensins on the production of macrophage proinflammatory cytokines, such as tumor necrosis factor-α (TNF-α) and interleukin-1β (IL-1β), and through the inhibition of IL-10 production[5].

Certain cytokines also have antimicrobial activity. Evaluation of chemokines for potential antimicrobial activity identified at least 17 of them that have antimicrobial effects[4]. For instance, macrophage inflammatory protein-3α (MIP-3α) exerts potent antimicrobial activity. Other inducible chemokines, including chemokine (C-X-C motif) ligands 9, 10, and 11 (CXCL9, CXCL10, CXCL11), and interferon-γ (IFN-γ), are active against *Escherichia coli* and *Listeria monocytogenes*[6]. Platelet basic protein (PBP) and other cytokines with antimicrobial properties were identified in the thrombin-activated human platelet[7]. Thus, in addition to their involvement in hemostasis, platelets contribute to innate immune function by delivering multifunctional chemokine-antimicrobial peptides to sites of vascular injury.

## ANTIMICROBIAL PROPERTIES OF NEUROPEPTIDES

The nervous system is a source of natural antimicrobial agents and a target for these molecules. Indeed, several peptides with neural or neuroendocrine signaling functions also have antimicrobial activity. This discovery suggests that the nervous system uses antimicrobial peptides as anti-infective agents by delivering them to innervated tissues[8].

An example of the capacity of the vertebrate nervous system to deliver antimicrobial peptides to sites of tissue injury is provided by the frog skin, which contains specialized neuroepithelial glands that synthesize and store antimicrobial peptides[9]. On adrenergic stimulation, these glands release their contents on the skin surface[10]. Therefore, the nervous system rapidly responds to noxious stimuli and conveys antimicrobial peptides to specific sites.

The capacity of the nervous system to protect epithelia through delivery of antimicrobial peptides is not restricted to amphibians. Indeed, several mammalian neuropeptides and hormones, including substance P, adrenomedullin, and proenkefalin A, have antimicrobial properties[8].

Recognized antimicrobial peptides, including defensins and cathelidicins, are produced within the central nervous system of many species[11,12,13,14]. Bovine β-defensin mRNA is expressed in the meninges, cortex, and Purkinje cells of the cerebellum[11]. Human β defensin-2 (HBD2) expression was induced in astrocytes incubated with lipopolysaccharide (LPS), IL-1-β, or TNF-α[12]. It appears, therefore, that antimicrobial peptides contribute to innate immunity within the brain.

These observations indicate that the bidirectional communication between the immune, neuroendocrine, and nervous systems based on common signal molecules and receptors[15] also include antimicrobial molecules[8]. Therefore, the neuroimmunomodulatory network, which protects the host against a variety of stressful challenges, comprises the capacity to reduce microbial invasion.

## α-MSH AS A NATURAL ANTIMICROBIAL PEPTIDE

α-Melanocyte stimulating hormone (α-MSH) is 13 amino acid anti-inflammatory peptide produced by many cell types including monocytes, endothelial cells, and keratinocytes[16]. The peptide is released locally and in the circulation on challenge to the host[17]. Blockade of the endogenous molecule with specific antibodies increased the febrile response to pyrogens in rabbits[18]; expression of proinflammatory mediators in the circulation, lungs, and liver of mice injected with endotoxin[19]; and release of TNF-α, IL-6, and nitric oxide by activated microglia *in vitro*[20]. It appears, therefore, that endogenous α-MSH contributes to control of host reactions. Administration of α-MSH in animal models of human disorders produces multiple beneficial effects on disease parameters[16,21,22].

The potential antimicrobial effects of α-MSH were explored based on the striking similarities of this anti-inflammatory molecule with natural antimicrobial peptides. Indeed, α-MSH appeared during the Paleozoic era, long before adaptive immunity developed and, similar to natural antimicrobial peptides, is produced by barrier epithelia[23,24,25], immunocytes[22], and within the central nervous system[26]. Data indicated that α-MSH and its C-terminal tripeptide Lys-Pro-Val have antimicrobial activity against two representative pathogens, *Staphylococcus aureus* and *Candida albicans*[27]. The candidacidal influences of α-MSH are exerted through a unique mechanism, substantially different from that of other natural antimicrobial peptides. α-MSH increased cyclic adenosine monophosphate (cAMP) production in *C. albicans* and the adenylyl cyclase inhibitor dideoxyadenosine (ddAdo) partly reversed the candidacidal effect of the peptide[27]. Of interest, both the cAMP inducer forskolin and the adenylyl cyclase inhibitor ddAdo alone caused some inhibition of colony-forming units. These data are consistent with recent observations that cAMP-activated pathways are of paramount importance for gene expression in *C. albicans*[28]. Therefore, it is reasonable to believe that the strong cAMP-inducing capacity of α-MSH interferes with the yeast's own regulatory mechanisms of this essential signaling pathway. Therefore, α-MSH effects in yeasts mimic peptide influences in mammalian cells in which α-MSH binds to G-protein-linked melanocortin receptors, activates adenylyl cyclase, and increases cAMP[29] (Fig.1).

The pathogenesis of *C. albicans* infection involves adhesion to host epithelial and endothelial cells and morphologic switching of yeast cells from the ellipsoid blastospores to various filamentous forms. α-MSH (1-13) and its C-terminal tripeptide α-MSH (11-13) greatly reduced germ tube formation of *C. albicans*[27]. Finally, α-MSH peptides did not reduce killing activity of neutrophils, but they rather enhanced it, likely as a consequence of the direct antimicrobial activity. This characteristic could be very important whenever the peptide be used to treat inflammation in an immunocompromised host.

Research on the antimicrobial effects of α-MSH indicated that the peptide also reduces viability of *S. aureus*[27] and kills several other Gram-positive and Gram-negative bacteria (including *Streptococcus*, *Pseudomonas,* and *Proteus* spp., unpublished observations). The broad spectrum of activity indicates further analogy with other natural antimicrobial molecules. The mechanism of the bactericidal effect of α-MSH is still unknown. Of interest, similar to defensins and cathelidicins, the antimicrobial activity of α-MSH and synthetic melanocortins decreases in the presence of high salt concentrations.

An interesting observation is that the staphylococcal exfoliative toxins A and B (ETA and ETB) cleave α-MSH[30]. ETA and ETB are 27-kDa esotoxins produced by strains of *Staphylococcus aureus* and are the causative agents of staphylococcal scalded-skin syndrome. The crystal structures of the ETs indicate that the proteins are members of the serine protease family of enzymes. Both toxins determine cleavage at specific glutamic acid residues of α-MSH. It is reasonable to believe that cleavage of α-MSH by ETA and ETB disrupts an important protective system in the skin.

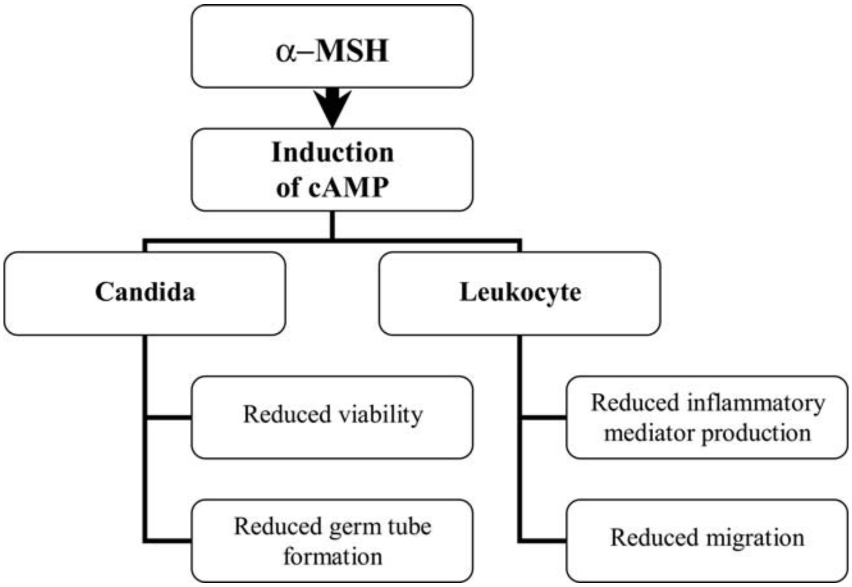

**FIGURE 1.** The candidacidal influences of α-MSH are exerted through induction of cAMP. Because cAMP-activated pathways are of paramount importance for gene expression in *C. albicans*, the strong cAMP-inducing capacity of α-MSH likely interferes with the yeast's own regulatory mechanisms of this essential signaling pathway. This mechanism mimics influences of the peptide in leukocytes and other cells in which α-MSH increases cAMP and modulates gene transcription.

## SYNTHETIC MELANOCORTINS WITH ANTIMICROBIAL PROPERTIES

The observation that α-MSH and its C-terminal tripeptide have antimicrobial properties prompted further research on synthetic derivatives that could be more suitable for clinical use[31]. The truncated peptide α-MSH (6-13), which had candidacidal activity similar to that of the full-length peptide α-MSH (1-13)[27], was used to design novel molecules. Further, the overlapping capacity of α-MSH to induce cAMP in mammalian and *C. albicans* cells suggested the presence of a melanocortin-like receptor(s) in yeast. Therefore, the design of synthetic analogues included amino acid substitutions known to alter melanocortin activity at the known melanocortin receptors. Substitution of $Phe^7$ with $D$$Phe^7$ or $D$$Nal^7$ markedly enhances potency of melanocortins in mammalian cells[32]. However, the increased activity linked to $D$$Phe^7$ substitution appears to be restricted to mammalian cells and does not occur in *C. albicans*. Conversely, $D$$Nal^7$ substitution caused a remarkable and very consistent increase in candidacidal activity. Activity was further enhanced when $D$$Nal^7$ was associated with the substitution of $Pro^{12}$ with $Phe^{12}$. This peptide killed 100% *C. albicans* on repeated experiments and was the most promising compound. The enhanced candidacidal activity of the $Phe^{12}$-substituted peptides was the most distinctive feature when compared to effects in mammalian cells[33]. Consistently, our experiments showed that the potent candidacidal peptide $D$$Nal^7$, $Phe^{12}$-α-MSH (6-13) had impaired anti-TNF-α properties in a rat model of endotoxemia[34]. Therefore, that candidacidal and anticytokine influences of melanocortins can be separated through selective amino acid substitutions to produce peptides that are primarily candidacidal or anti-cytokine. Conversely, it is possible to obtain synthetic melanocortins that combine antimicrobial and anti-inflammatory effects.

The C-terminal tripeptide Lys-Pro-Val (α-MSH 11-13) exerts anti-inflammatory influences similar to those of the parent molecule and shows substantial candidacidal influences. Therefore, this short molecule formed the basis for novel small melanocortin molecules with candidacidal properties. The dimer $(CKPV)_2$ obtained by inserting a Cys-Cys linker between two units of Lys-Pro-Val-$NH_2$ showed excellent candidacidal effects against *Candida* spp., including strains of *C. krusei* and *C. glabrata*[35] that are often resistant to current candidacidal agents. In addition to the candidacidal effects, $(CKPV)_2$ also exerted anti-inflammatory influences similar to those of the parent molecule[36]. This molecule is presently evaluated in clinical trials in the U.S. and in Europe, and should soon be available for clinical use.

Because of its potent candidacidal influences, $(CKPV)_2$ could represent a scaffold to design further antimicrobial compounds. Therefore NMR spectroscopy was used to determine the tertiary structure of the peptide[35]. $(CKPV)_2$ appeared to be a symmetric dimer that assumes an extended backbone structure in which all the amide bonds adopt preferentially a *trans* conformation; further, the peptide follows a β-turn-like structure at the $Pro^3$-$Val$-$NH_2^4$ level. Of interest, this structure shows similarities with the conformation adopted by α-MSH[37].

## CONCLUSIONS

The ancient pleiotropic peptide α-MSH contributes to the host defense by exerting antimicrobial effects. When considering that most of the natural antimicrobial peptides enhance inflammatory reactions, the anti-inflammatory and antipyretic effects of α-MSH confer unique properties to this molecule that could be very beneficial. Synthetic derivatives, chemically stable and resistant to enzymatic degradation, could form the basis for novel therapies that combine anti-inflammatory and antimicrobial properties.

## ACKNOWLEDGMENTS

This work was supported by Programma di Ricerca Scientifica di Rilevante Interesse Nazionale (PRIN) 2004 “Biomolecular evaluation of novel melanocortin peptides and peptidomimetics with antifungal properties”.
